# Novel Nanocomposite Inhibiting Caries at the Enamel Restoration Margins in an In Vitro Saliva-Derived Biofilm Secondary Caries Model

**DOI:** 10.3390/ijms21176369

**Published:** 2020-09-02

**Authors:** Wen Zhou, Xinyu Peng, Xuedong Zhou, Andrea Bonavente, Michael D. Weir, Mary Anne S. Melo, Satoshi Imazato, Thomas W. Oates, Lei Cheng, Hockin H. K. Xu

**Affiliations:** 1 State Key Laboratory of Oral Diseases, Department of Operative Dentistry and Endodontics, West China School of Stomatology, National Clinical Research Centre for Oral Diseases, Sichuan University, Chengdu 610041, China; zhouwendentist@139.com (W.Z.); pengxinyu@stu.scu.edu.cn (X.P.); zhouxd@scu.edu.cn (X.Z.); 2 Department of Advanced Oral Sciences and Therapeutics, University of Maryland Dental School, Baltimore, MD 21201, USA; andreamarie899@gmail.com (A.B.); MWeir@umaryland.edu (M.D.W.); MMelo@umaryland.edu (M.A.S.M.); toates@umaryland.edu (T.W.O.); 3 Fujian Key Laboratory of Oral Diseases & Fujian Provincial Engineering Research Center of Oral Biomaterial & Stomatological Key Lab of Fujian College and University, School of Stomatology, Fujian Medical University, Fuzhou 350002, China; 4 Department of Biomaterials Science, Osaka University Graduate School of Dentistry, Osaka 565-0871, Japan; imazato@dent.osaka-u.ac.jp; 5 Center for Stem Cell Biology & Regenerative Medicine, University of Maryland School of Medicine, Baltimore, MD 21201, USA; 6 Marlene and Stewart Greenebaum Cancer Center, University of Maryland School of Medicine, Baltimore, MD 21201, USA

**Keywords:** secondary caries, remineralization, antibacterial, nanocomposite, saliva-derived biofilms, enamel hardness

## Abstract

Secondary caries often occurs at the tooth-composite margins. This study developed a novel bioactive composite containing DMAHDM (dimethylaminohexadecyl methacrylate) and NACP (nanoparticles of amorphous calcium phosphate), inhibiting caries at the enamel restoration margins in an in vitro saliva-derived biofilm secondary caries model for the first time. Four composites were tested: (1) Heliomolar nanocomposite, (2) 0% DMAHDM + 0% NACP, (3) 3% DMAHDM + 0% NACP, (D) 3% DMAHDM + 30% NACP. Saliva-derived biofilms were tested for antibacterial effects of the composites. Bovine enamel restorations were cultured with biofilms, Ca and P ion release of nanocomposite and enamel hardness at the enamel restoration margins was measured. Incorporation of DMAHDM and NACP into composite did not affect the mechanical properties (*p* > 0.05). The biofilms’ CFU (colony-forming units) were reduced by 2 logs via DMAHDM (*p* < 0.05). Ca and P ion release of the nanocomposite was increased at cariogenic low pH. Enamel hardness at the margins for DMAHDM group was 25% higher than control (*p* < 0.05). With DMAHDM + NACP, the enamel hardness was the greatest and about 50% higher than control (*p* < 0.05). Therefore, the novel composite containing DMAHDM and NACP was strongly antibacterial and inhibited enamel demineralization, resulting in enamel hardness at the margins under biofilms that approached the hardness of healthy enamel.

## 1. Introduction

Dental composites are increasingly popular for restoring carious teeth due to their excellent aesthetics. Secondary caries refers to caries at the margins of the existing restorations [1,2,3]. Composite polymerization shrinkage and degradation of the bonding interface could result in micro-gaps at the margins between the composite and the tooth structures [4,5,6,7]. Microleakage of bacteria and their acid by-products through these gaps is the main reason for secondary caries [8]. Therefore, suppressing the bacteria and reducing their acid production are important in inhibiting caries. Hence, efforts have been made to develop antibacterial composites to combat the bacteria at the margins of the restorations [9,10]. Quaternary ammonium methacrylates (QAMs) can copolymerize to dental methacrylate monomers in the resins, thus achieving long-term antibacterial effects [11,12,13,14,15,16,17,18]. Previous studies developed 12-methacryloyloxydodecylpyridinium bromide (MDPB), quaternary ammonium polyethylenimine (QPEI) nanoparticles, quaternary ammonium methacryloxy siliane molecule (QAMS; C_44_H_90_ClNO_18_Si_5_), methacryloxylethyl cetyl dimethyl ammonium chloride (DMAE-CB), 2-dimethyl-2-dodecyl-1-methacryloxyethyl ammonium iodine (DDMAI) and novel quaternary ammonium dimethacrylate monomers (IQM) [11,12,13,14,15,16]. These antibacterial agents were incorporated into dental resins with potent and broad-spectrum antibacterial effects [11,12,13,14,15,16]. Recently, resins containing a new antibacterial monomer, dimethylaminohexadecyl methacrylate (DMAHDM), were proven to have a strong antibacterial activity [19,20]. However, none of these previous studies investigated the ability of antibacterial composites to combat enamel demineralization around the restorations under biofilm acid attacks.

Calcium phosphate particles were used as ion-releasing fillers in resins [21,22]. Calcium (Ca) and phosphate (P) ions from the resin could make the surrounding medium supersaturated with these ions, thus inhibiting demineralization and promoting remineralization [23]. Recently, nanoparticles of amorphous calcium phosphate (NACP) with a mean size of 116 nm were synthesized [21,24]. Weir et al. have demonstrated that NACP composite remineralized the demineralized enamel and dentin using in vitro chemical demineralization and remineralization cycles [21,25]. Liang et al. achieved dentin remineralization via NACP composite in acid challenge environment or even in acidic solution without additional Ca and P ions [26,27]. Furthermore, NACP nanocomposite inhibited caries formation in a human in situ model [28].

Therefore, a composite containing both DMAHDM and NACP would be highly promising for inhibiting secondary caries. Biofilm-based in vitro secondary caries models enable the investigation of several key variables in a relatively simple, fast, and cost-effective manner when compared to in situ and clinical human trial models [29]. Li et al. challenged the dentin-composite samples with an in vitro saliva-derived biofilm model for 3 days [30]. Scanning electron microscopy and energy-dispersive X-ray spectroscopy were used to assess the demineralization and interfacial degradation of the dentin-restoration fracture surfaces. The results showed that secondary caries was successfully induced around the restorations [30]. Previous studies demonstrated that stable microcosm oral biofilms can be produced from human saliva samples to test the properties of restorative materials or the generation and progression of secondary caries [30,31]. Kuper et al. investigated the effects of restorative materials and adhesives on secondary caries formation using an in vitro saliva-derived biofilm model [32]. They demonstrated that Clearfil AP-X composite (Kuraray Medical Inc., Okayama, Japan) with Protect Bond (Kuraray) limited the advanced secondary caries lesion formation when compared to other five commercial restorative materials and adhesives [32]. Therefore, it would be reasonable to use a saliva-derived biofilm model with artificial saliva medium to investigate the secondary caries prevention effects of the novel composite containing DMAHDM and NACP.

Secondary caries occurs at the tooth-restoration margins, where the cavity wall meets the restoration [33]. Ideally, the prevention of secondary caries should begin at the time of restoration placement [34]. At the initial stage of secondary caries, the demineralization can be arrested by remineralization, and these lesions are limited in micron scales around the restorations [35,36]. In addition, the enamel hardness mapping adjacent to the restoration can reveal information on the demineralization level of enamel around the margin and show the potential of demineralization prevention of the restorative material [37]. Therefore, enamel hardness measurement was performed for investigating the demineralization inhibition capability of the composite restorative.

Therefore, the objectives of this study were to: (1) develop a DMAHDM + NACP composite and investigate its antibacterial effects against saliva-derived biofilm and Ca and P ion release, and (2) determine the effects on enamel demineralization and hardness at the margins under saliva-derived biofilms for the first time. It was hypothesized that: (1) The DMAHDM composite would reduce the cariogenic abilities of saliva-derived biofilm and preserve the enamel hardness around the restoration margins under biofilms, when compared to control group; (2) The combination of DMAHDM and NACP in the composite would inhibit biofilm cariogenic abilities while providing Ca and P ions; (3) The DMAHDM + NACP composite would protect the restoration margins and yield the greatest enamel hardness at the margins under biofilm acid attacks among all the groups tested.

## 2. Results

**Figure 1:**
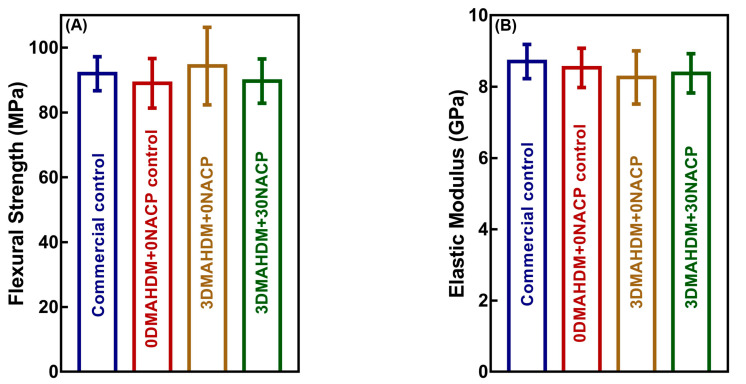
Mechanical properties of composites. (**A**) Flexural strength, and (**B**) elastic modulus (mean ± SD; *n* = 6). There was no significant difference among the four groups (*p* > 0.05).

The mechanical properties of the composites are plotted in Figure 1 (mean ± standard deviation (SD); *n* = 6). Compared to commercial control and 0DMAHDM + 0NACP, the incorporation of 3% DMAHDM and 30% NACP had no adverse effect on flexural strength and elastic modulus (*p* > 0.05).

**Figure 2:**
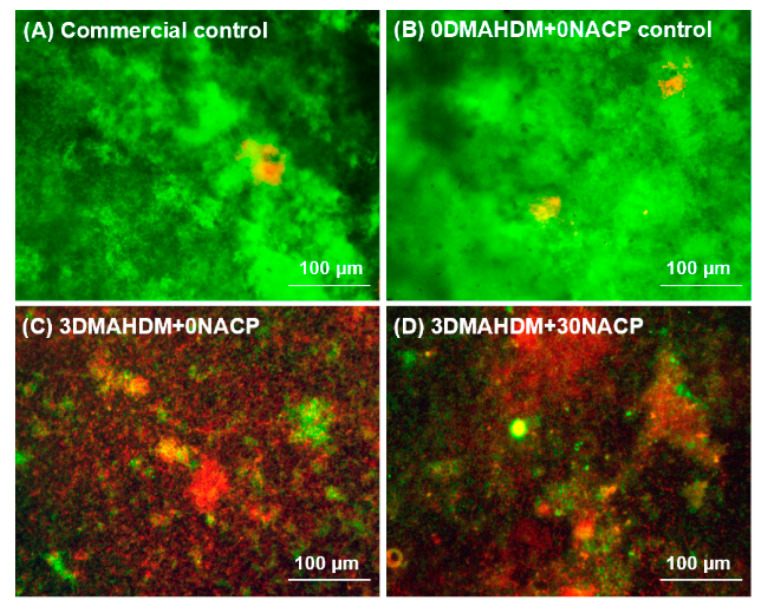
Representative images of live/dead stained biofilms grown for 48 h on composites. (**A**) Commercial control. (**B**) 0DMAHDM + 0NACP control. (**C**) 3DMAHDM + 0NACP. (**D**) 3DMAHDM + 30NACP. Live bacteria were stained green and dead bacteria were stained red. Commercial control and 0DMAHDM + 0NACP control composites had primarily live bacteria. 3DMAHDM + 0NACP and 3DMAHDM + 30NACP produced mostly red staining.

Figure 2 shows representative live/dead images of the saliva-derived biofilms on the composites. The commercial control and 0DMAHDM + 0NACP were covered by live bacteria. In contrast, 3DMAHDM + 0NACP and 3DMAHDM + 30NACP had substantial amounts of compromised bacteria with red staining.

**Figure 3:**
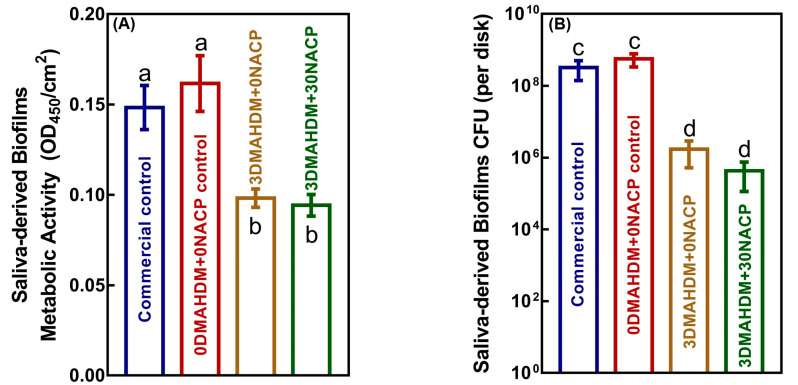
Antibacterial effects of composites on saliva-derived biofilm. (**A**) MTT metabolic activity and (**B**) colony-forming units (CFU) of saliva-derived biofilms on composites (mean ± SD; *n* = 6). Bars with disparate letters indicate data are significantly different (*p* < 0.05).

As shown in Figure 3A, 3DMAHDM + 0NACP and 3DMAHDM + 30NACP yielded much lower metabolic activity of biofilms than commercial control and 0DMAHDM + 0NACP control (*p* < 0.05). Figure 3B plots CFU (colony-forming unit) counts of the 48 h saliva-derived biofilms for each group (mean ± SD; *n* = 6). The two control composites had the highest CFU counts. Compared to control, both 3DMAHDM + 0NACP and 3DMAHDM + 30NACP composites reduced the saliva-derived biofilm CFU by two orders of magnitude (*p* < 0.05). These results demonstrate that DMAHDM exerted potent antibacterial effects. Further incorporation of NACP did not influence the antibacterial properties of the composite. The 3DMAHDM + 30NACP composite had similar biofilm inhibition abilities to 3DMAHDM + 0NACP composite.

**Figure 4:**
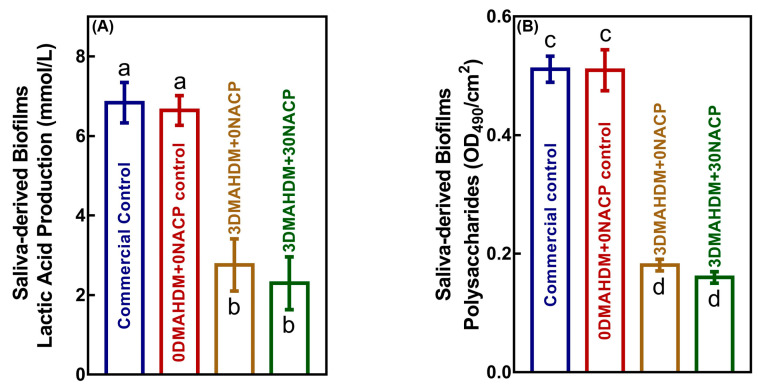
Inhibition effects of composites against cariogenic activities of saliva-derived biofilms (mean ± SD; *n* = 6). (**A**) Lactic acid production by saliva-derived biofilms. (**B**) Polysaccharide production by saliva-derived biofilms on composites. Values with dissimilar letters are significantly different from each other (*p* < 0.05).

Lactic acid and polysaccharide production of 48 h saliva-derived biofilms on composites are plotted in Figure 4A,B, respectively (mean ± SD; *n* = 6). The biofilms on the two control composites produced the most acid and polysaccharides. The composites with 3DMAHDM + 0NACP and 3DMAHDM + 30NACP substantially reduced the acid and polysaccharide production of the saliva-derived biofilms (*p* < 0.05). These results demonstrate that 3DMAHDM + 0NACP and 3DMAHDM + 30NACP composite diminished the cariogenic abilities of the saliva-derived biofilms.

**Figure 5:**
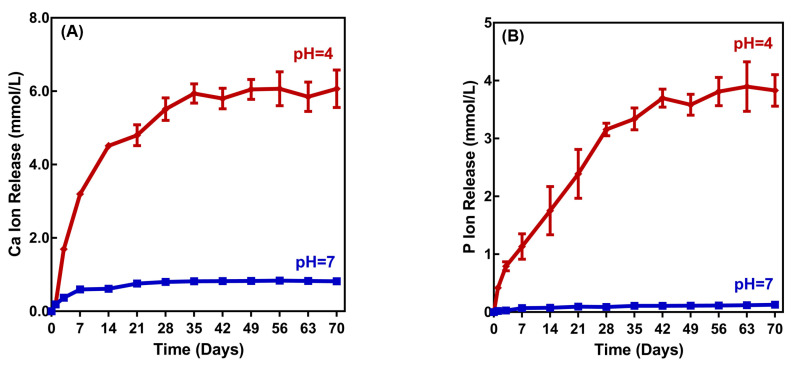
Calcium (Ca) and phosphate (P) ion releases from the 3DMAHDM + 30NACP composite immersed in solutions of pH 4 and 7. (**A**) Ca ion release, and (**B**) P ion release.

The Ca and P ion releases are plotted in Figure 5 for the composite with 3DMAHDM + 30NACP. When the pH of the solution decreased from 7 to 4, the ion release significantly increased. At 28 days and pH 4, the Ca ion release was (5.51 ± 0.53) mmol/L, much higher than (0.80 ± 0.04) mmol/L at pH 7. These results demonstrate that the ion release of 3DMAHDM + 30NACP composite “smartly” increased dramatically at a cariogenic pH of 4, when such ions would be most needed to combat tooth demineralization.

**Figure 6:**
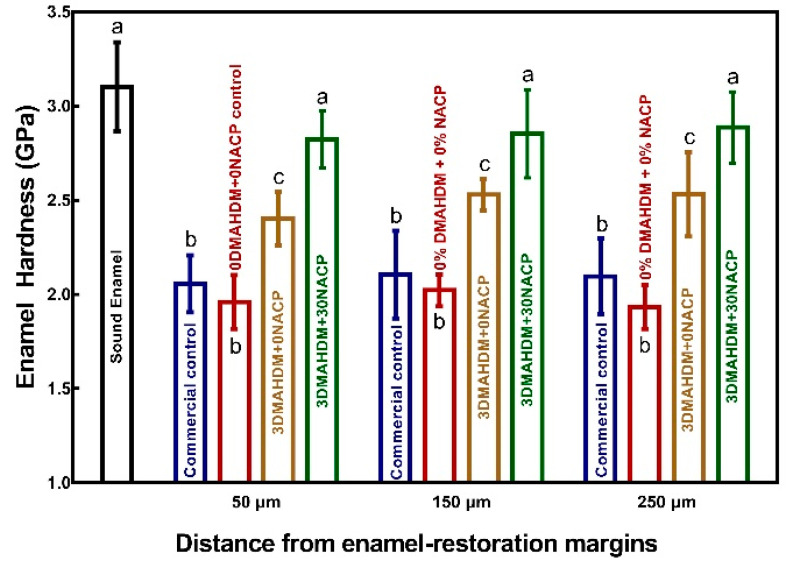
Enamel hardness for the four groups at three distances from the composite-enamel margins after saliva-derived biofilms acid attack with sucrose for 21 days (mean ± SD; *n* = 6). Values with dissimilar letters are significantly different from each other (*p* < 0.05).

The enamel hardness at the margins is plotted in Figure 6 (mean ± SD; *n* = 6). The hardness of sound enamel (without biofilm acid attack) was 3.10 ± 0.23 GPa. The enamel hardness at different distances from the interface showed that after the saliva-derived biofilm acid attack, the enamel hardness of commercial control and 0DMAHDM + 0NACP both decreased (*p* < 0.05). However, the enamel hardness around the 3DMAHDM + 0NACP was higher than the two control groups (*p* < 0.05). The 3DMAHDM + 30NACP group had the highest enamel hardness, which was not significantly different from that of healthy enamel (*p* > 0.05). For example, at a distance of 50 μm, the enamel hardness for commercial control and 0DMAHDM + 0NACP control was 2.05 ± 0.15 GPa and 1.96 ± 0.35 GPa, respectively. In contrast, the enamel hardness was 2.82 ± 0.15 GPa for 3DMAHDM + 30NACP, statistically similar to 3.10 ± 0.23 GPa of sound enamel (*p* > 0.05). These results demonstrate that the 3DMAHDM + 0NACP composite reduced enamel demineralization under saliva-derived biofilm acid attacks; furthermore, the combination of 3% DMAHDM and 30% NACP caused synergistic effects and further protected the enamel hardness.

## 3. Discussion

The present study used an in vitro secondary caries model with saliva-derived biofilms and demonstrated that the bioactive DMAHDM + NACP nanocomposite inhibited enamel demineralization and protected enamel hardness at the margins for the first time. The hypotheses were proven that the composite containing DMAHDM alone reduced the cariogenicity of saliva-derived biofilm and protected enamel hardness around the restoration margins under biofilms. Furthermore, the composite containing DMAHDM + NACP had even better protection for the restoration margins, resulting in the greatest enamel hardness at the margins under biofilm acid attacks among all the groups tested. For the DMAHDM + NACP group, the enamel hardness at the margins under biofilms for 21 days was not significantly different from the hardness of healthy enamel control. Therefore, the bioactive DMAHDM + NACP composite is promising for tooth cavity restorations to inhibit secondary caries at the margins and protect the tooth structures.

Secondary caries often occur in enamel along the tooth restoration margins [38]. The enamel along the margins will demineralize if the local conditions change to an acidic environment [29]. When the demineralization caused by bacterial acid is too severe to be reversed by natural remineralization via saliva, secondary caries will occur [39,40]. Therefore, it is highly desirable to develop a new generation of bioactive and therapeutic composites that can inhibit biofilm growth and possess remineralization capabilities [41]. In the present study, 3% DMAHDM in the composite substantially reduced the saliva-derived biofilm metabolic activity, lactic acid and polysaccharide production, and reduced the biofilms’ CFU by two orders of magnitude. DMAHDM possesses the positively charged quaternary amine N+ that can interact with the negatively charged cell membrane of the bacteria, leading to membrane disruption and cytoplasmic leakage [42,43]. In addition to the charge density, DMAHDM has an alkyl amine chain length of 16 which was postulated to be able to be inserted into the bacterial membrane, leading to physical disruption and bacterial death [42,43]. DMAHDM can be copolymerized with resin to be immobilize in the polymer, thus providing long-term antimicrobial ability [43]. Its stability has been demonstrated even after 180 days water-aging, with no significant decrease in the strong anti-biofilm activity of the DMAHDM-containing composite after aging [44]. Furthermore, DMAHDM-containing resins has a good biocompatibility, similar to commercial dental resins already used clinically [45]. These previous studies indicate that DMAHDM has the potential to be compatible with oral tissues and suitable for use in resins for clinical applications.

When using DMAHDM alone in the composite, the demineralization of enamel at three different distances from the enamel restoration interface was substantially reduced by the 3DMAHDM + 0NACP under saliva-derived biofilm acid attack, yielding enamel hardness that was 25% higher than that of the 0DMAHDM + 0NACP control. In the biofilms on the composite, endogenous bacteria produce organic acids as a by-product of metabolism of fermentable carbohydrates. This acid causes the local pH to fall, resulting in demineralization of enamel [46]. Polysaccharides produced by cariogenic bacteria in biofilms act as carbohydrate reservoirs, with a matrix scaffold to promote bacterial adherence and as a barrier to protect the bacteria from endogenous or exogenous antimicrobial factors [47,48,49,50]. It can be speculated that the DMAHDM composite disrupted the saliva-derived biofilm formation, viability and cariogenic activities, thus shifting the local ecosystem to a less cariogenic state. This in turn decreases the extent of demineralization of enamel around the restoration when compared with the control group.

In addition, when DMAHDM and NACP were both incorporated into the composite, the efficacy of protecting the teeth was further increased [51]. The incorporation of 30% NACP did not negatively influence the mechanical properties of the composite when compared to the control composite. The nano-size of NACP and the reinforcement by the glass filler particles enabled the stability of the mechanical properties of the composite. Indeed, Cheng et al. demonstrated that the flexural strength and elastic modulus of a composite containing NACP and QAM were similar to those of a commercial control composite (Renamel, Cosmedent, Chicago, IL, USA) both before and after one-year water-aging treatment [52]. In the present study, both DMAHDM and NACP contributed to the caries inhibition effectiveness under saliva-derived biofilms, and resulted in the greatest enamel hardness, which statistically matched the hardness of healthy enamel. The following reasons contributed to the inhibition of secondary caries by the DMAHDM + NACP nanocomposite. First, DMAHDM in the composite had potent antibacterial capability, hence the saliva-derived biofilm acids were greatly reduced. Second, the NACP composite was “smart” and released high levels of Ca and P ions at the cariogenic pH, when these ions were most needed to inhibit caries. The 3DMAHDM + 30NACP composite had little ion release at pH 7, thus preserving the ion reservoir when these ions were not needed. However, when the pH decreased and the ions were needed to promote remineralization, the composite greatly increased the ion release at pH 4. Such ion release could neutralize the acid produced by the residual bacteria, thus increasing the local plaque pH to avoid demineralization of the enamel. Indeed, previous studies showed that NACP composite could neutralize the acid and raise the pH from a cariogenic pH 4 to a safe pH of 6 to 7 [53,54]. Third, the Ca and P ion release from NACP increased the ion concentration around the restoration margins, thereby tilting the balance toward remineralization [21,25]. Collectively, the alteration of the local bacterial ecosystem, the higher local pH, and the remineralizing chemical environment provided by the 3DMAHDM + 30NACP composite successfully inhibited secondary caries at the margins.

Secondary caries models have been used in testing agents, materials and techniques that inhibit demineralization or promote remineralization [29,55]. The advantages of in vitro models are the better control of the test in obtaining results versus in situ or in vivo models that require volunteers to perform the study [56]. Compared to chemical demineralization methods that were previously used [21,25,26,27], the present saliva-based biofilm model was able to measure the secondary caries inhibition effects of composites at the enamel restoration margins under biofilms, which better replicated the clinical conditions. The following three aspects should be noted. First, human saliva and sucrose were commonly used to establish in vitro dental plaque biofilm secondary caries models [23,57,58]. Microcosm biofilms from human saliva are similar to natural plaque and overcome many difficulties encountered in studying in vivo biofilms, such as lack of standardization among subject characteristics [59]. The saliva was donated from ten donors in the present study which was then mixed to have the diversity and heterogeneity for the biofilm model. Second, the McBain medium is an artificial saliva medium which could increase the carbohydrate metabolism of the saliva-derived biofilms, thus was suitable for the present study [60]. In this saliva-derived biofilm model, 0.2% sucrose was added into the McBain medium. This concentration of sucrose in the medium was confirmed previously to have the capability to produce acidic pH due to the polymicrobial biofilm growth and acid production [58,61]. Third, the buffering effect of PIPES in the McBain medium was similar to that of the buffer system consisting of bicarbonate and phosphate in the whole saliva. Therefore, the present study used a clinically relevant biofilm-based secondary caries model.

Under these conditions, with the combination of anti-biofilm effects of DMAHDM and Ca and P ions from NACP, the enamel hardness in the 3DMAHDM + 30NACP group showed a minimal decrease at the enamel restoration margin under 21 days of biofilm acid attack with a constant sucrose presence. These results demonstrate that this bioactive composite can effectively inhibit demineralization in the margins, prevent secondary caries and protect tooth structures. Further studies are needed to investigate biofilm attacks and the effect of DMAHDM + NACP composite-tooth restoration samples under biofilms for longer than 21 days on marginal enamel hardness. Further studies are also needed to investigate the DMAHDM + NACP method for applications in dental adhesives, cements and sealants via biofilm-based caries models on the protection of enamel and dentin hardness.

## 4. Materials and Methods

### 4.1. Fabrication of Composites

Bisphenol glycidyl dimethacrylate (BisGMA, Esstech, Essington, PA, USA) and TEGDMA (Esstech, Essington, PA, USA) were mixed at a mass ratio of 1:1 [62,63], and rendered light-curable with 0.2% camphorquinone and 0.8% ethyl 4-N, Ndimethylaminobenzoate. This photo-activated BisGMA-TEGDMA resin was referred to as the BT resin [64].

DMAHDM with an alkyl chain length of 16 was synthesized using a modified Menschutkin reaction where a tertiary amine group was reacted with an organo-halide. A benefit of this reaction was it was done at virtually quantitative amounts and required minimal purification [65]. NACP was formed via a spray-drying technique [24]. This method produced NACP with a mean size of 116 nm [24]. Barium boroaluminosilicate glass particles with a median size of 1.4 μm (Caulk/Dentsply, Milford, DE, USA) were used as a co-filler for mechanical reinforcement. The glass particles were silanized with 4% 3-methacryloxypropyltrimethoxysilane [66,67]. For the fabrication of composite with DMAHDM and NACP, 35% glass particles, 32% BT and 3% DMAHDM were mixed with 30% NACP. These mass fractions were selected following a previous study [64]. The novel composites were tested and confirmed to possess stable mechanical properties. In addition, Heliomolar (Ivoclar, Amherst, NY, USA), a fluoride-releasing composite served as a control. It contained 40–200 nm nano-silica and ytterbium-trifluoride at a filler mass fraction of 66.7%. Therefore, the following four composites were tested:

(1) Heliomolar nanocomposite (referred to as Commercial control);
(2) Experimental composite control. 35% BT + 65% glass particles (referred to as 0DMAHDM + 0NACP control);
(3) Antibacterial composite. 32% BT + 65% glass particles + 3% DMAHDM + 30% NACP (referred to as 3DMAHDM + 0NACP);
(4) Antibacterial and remineralizing composite. 32% BT + 35% glass particles + 3% DMAHDM + 30% NACP (referred to as 3DMAHDM + 30NACP).

### 4.2. Mechanical Testing

Each composite paste was placed into a rectangular mold of 2 × 2 × 25 mm. The sample was covered in Mylar strips and light-cured (Triad 2000; Dentsply, York, PA, USA) for 1 min on each open side of the mold [68,69]. The specimens were put in water at 37 °C for 24 h. Three-point flexural strength was tested on a computer-controlled Universal Testing Machine (5500R, MTS, Cary, NC, USA) at a crosshead speed of 1 mm/min [70]. Six specimens were evaluated for each group (*n* = 6). Flexural strength was measured with the following formula: S = 3P_max_/L(2bh^2^), where P_max_ is the fracture load, L is span, b is specimen width and h is thickness. Elastic modulus was determined by: E = (P/d)(L^3^/[4bh^3^]), where P is the load, d is the corresponding displacement.

### 4.3. Composite Disk Preparation for Biofilm Tests

Molds with a diameter of 9 mm and a thickness of 1 mm were used to prepare the composite disks. Each open side of the disk was covered with a Mylar strip, and the sample was light-cured (Triad 2000; Dentsply, York, PA, USA) for 1 min. The disks were subjected to immersion in 200 mL of deionized water with stirring at 100 rpm for 1 h to remove the initial burst of any small amounts of un-polymerized monomers [71]. Then sterilization for the samples was done with ethylene oxide (Anprolene AN 74i, Andersen, Haw River, NC, USA). The degassing of the disks was performed for 7 days.

### 4.4. Bacterial Culture and Biofilm Formation on Composites

The use of the dental plaque microcosm biofilms with human saliva as inoculum was approved by University of Maryland Baltimore Institutional Review Board. Ten healthy volunteers were chosen as donors who had natural dentition and without periodontal disease and active caries, who did not take any antibiotics in the last 3 months. An identical volume of saliva from each donor was pooled together and diluted 2-fold with sterile 50% glycerol, which was stored at −80 °C as described in a previous study [72].

A McBain artificial saliva medium (1.5 mL) was added to each well of 24-well plates with a composite disk. The medium consisting of 2.5 g/L mucin, 2.0 g/L Bacto peptone, 2.0 g/L Trypticase peptone, 1.0 g/L yeast extract, 0.35 g/L NaCl, 0.2 g/L KCl, 0.2 g/L CaCl_2_, 0.001 g/L hemin, and 0.0002 g/L vitamin K1, with 0.2% sucrose and 50 mmol/L PIPES at pH 7.0 [55]. The saliva-glycerol stock was added with 1:50 final dilution. To better replicate conditions existing in supragingival plaque, the saliva-derived biofilms were incubated in aerobic environment, 5% CO_2_ at 37 °C [31]. After 24 h, the composite disks with biofilms were transferred to new 24-well plates with fresh medium, and incubated for another 24 h [73]. This totaled a culture time of 48 h, which was sufficient to form microcosm biofilms on the resins [72].

### 4.5. Live/Dead Bacterial Assay

The composite disks with 48 h biofilms were washed three times with cysteine peptone water (CPW) to remove any non-adherent bacteria. Three specimens per group were stained with BacLight live/dead bacterial viability kit (Molecular Probes, Eugene, OR, USA) [67]. Each specimen was photographed in five randomly-selected fields of view. Live bacteria were stained with SYTO 9 to produce a green fluorescence. The compromised bacteria were stained with propidium iodide to produce a red fluorescence. An inverted epifluorescence microscope (TE2000-S, Nikon, Melville, NY, USA) was used to image the biofilms.

### 4.6. Biofilms CFU Counts

Separate specimens were prepared for biofilm CFU measurement. After washing three times in PBS, the disks with 48 h biofilms were transferred into tubes with 1 mL of CPW. The biofilms were harvested by sonication (3510R-MTH, Branson, Danbury, CT, USA) and vortexing (Fisher, Pittsburgh, PA, USA) according to previous studies [64,72]. The suspensions were serially diluted and spread on tryptic soy blood agar culture plates. Six disks were tested for each group. After 48 h incubation at 37 °C in 5% CO_2_, the colony number was counted and CFU counts were determined [66].

### 4.7. MTT Metabolic Assay of Biofilms

MTT assay is a method of detecting cell survival and growth. This assay relies on the active bacterial metabolization of 3-(4,5-dimethylthiazol-2-yl)-2,5-diphenyltetrazolium bromide (MTT), a yellow tetrazolium salt, to form a purple formazan crystal in metabolically active cells [52]. Composite disks were inoculated with bacteria and cultured for 48 h as described in Section 4.4. The biofilms were washed twice with PBS and placed in 24-well plates (*n* = 6), and inoculated with 1 mL of MTT solution (with 0.5 mg/mL MTT in PBS) for 1 h at 37 °C in 5% CO_2_ [64]. Then, the disks were transferred into new 24-well plates, and 1 mL of dimethyl sulfoxide (DMSO) was added to dissolve the formazan crystals. The plates were incubated for 20 min with gentle mixing. Two hundred microliters of the DMSO solution was then transferred into 96-well plates, and OD_540 nm_ was determined using a microplate reader (SpectraMax M5, Molecular Devices, Sunnyvale, CA, USA) [64].

### 4.8. Lactic acid Production by Biofilms

Enzymatic lactate analysis was used to examine the lactic acid production by biofilms. The 48 h biofilms were washed twice with PBS. The disks with biofilms were immersed in 1.5 mL buffered peptone water (BPW) plus 0.2% sucrose and incubated at 37 °C in 5% CO_2_ for 3 h (*n* = 6). The BPW solutions were then collected to analyze the lactate content using an enzymatic method [72]. The OD_340 nm_ of the BPW was measured using a microplate reader (SpectraMax M5, Molecular Devices, Sunnyvale, CA, USA). Standard curves were prepared using a standard lactic acid (Supelco, Bellefonte, PA, USA) [64].

### 4.9. Water-Insoluble Polysaccharide Production by Biofilms

Water-insoluble polysaccharides of the biofilms were measured using a phenol-sulfuric acid method [20]. The 48 h biofilms on disks were collected by scraping and sonication/vortexing in 2 mL PBS, followed by centrifugation at 12,000 rpm for 3 min (Eppendorf Centrifuge 5415, Brinkmann, Westbury, NY, USA) (*n* = 6). The precipitate was then washed twice with PBS and resuspended in 200 μL of distilled water. Then, 200 μL of 5% phenol solution and 1 mL of 95–97% sulfuric acid were added, and the samples were incubated for 30 min. Then, 200 μL of the solution was transferred into a 96-well plate and the OD_490nm_ was determined using the microplate reader [20].

### 4.10. Ca and P Ion Release

Next, 50 mmol/L lactic acid and 50 mmol/L HEPES were used to buffer the sodium chloride (NaCl) solution (133 mmol/L) into pH 4 and pH 7, respectively. Three specimens of 2 × 2 × 12 mm were submerged into 50 mL of a solution at each pH. This produced a specimen volume to solution ratio of 2.9 mm^3^/mL, which was similar to the previous specimen volume to solution ratio of approximately 3.0 mm^3^/mL [74]. The concentrations of Ca and P were measured at 1, 3, 7, 14, 21, and 28 days. At each time, an aliquot of 0.5 mL was removed and replaced with a fresh solution. The pH of the solution was checked and adjusted to pH 4 using the 50 mmol/L of lactic acid. The aliquots were analyzed for Ca and P ions using a spectrophotometric method (DMS-80 UV-visible, Varian, Palo Alto, CA, USA) with known standards and calibration curves [74].

### 4.11. Saliva-Derived Biofilm Model for Enamel Demineralization at the Margins

**Figure 7:**
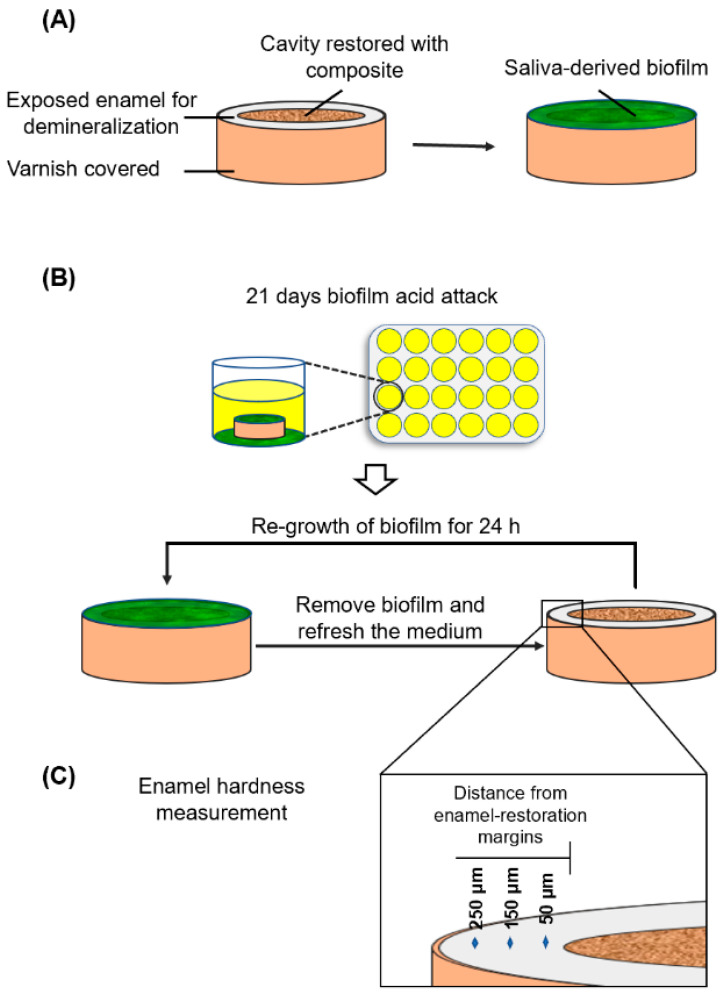
Schematic diagram illustrating the workflow of establishing saliva-derived biofilm secondary caries model. (**A**) Enamel slabs were restored with composites. Saliva-derived biofilm was formed on the specimens. (**B**) 21-day biofilm acid attack. (**C**) Enamel hardness measurement at different distances from the enamel restoration margins.

The use of freshly extracted bovine teeth was approved by University of Maryland Baltimore Institutional Review Board. Twenty-four bovine incisors were used to prepare 24 enamel slabs with a diameter of 6 mm. Circular cavities with an approximate diameter of 4 mm and a depth of 1.5 mm were prepared. A circle of 1 mm width of enamel surface area around the cavity was exposed. The rest of the enamel surfaces were covered with two layers of acid-resistant nail varnish (Figure 7A). For each group, 6 slabs were restored with the respect composite. In order to isolate and determine the effects of the composite without interference from an adhesive, no adhesive was used in the present study [75]. After 24 h, the specimens were polished with sandpapers using grit of # 600, 1200, 2400 and 4000 consecutively. As shown in Figure 7B, the enamel slabs were placed into a 24-well plate. The saliva-glycerol stock was added with 1:50 final dilution in 1.5 mL of McBain into each well. The medium was refreshed and the biofilms on the slab were removed by soft sterilized paper every 24 h. This was done to simulate the daily oral hygiene activities that lead to the removal and re-growth of the dental biofilms, and because relatively mature biofilms can be formed from the saliva samples in 24 h [76]. The slab was placed into the well and inoculated to re-grow the biofilms for 1 day. This process was repeated for 21 days as described above. Previous studies showed that 21 days of saliva-derived biofilm exposure was able to induce secondary caries lesions around the restorations [29,32].

### 4.12. Hardness Measurement of Enamel at the Margins

After 21 days of biofilm acid attack, Vickers hardness was measured on enamel surface near the enamel composite margin (Figure 7C). Three different distances from the interface of the cavity were chosen for hardness measurement: 50 μm, 150 μm, 250 μm, following a previous study [77]. Vickers indentation (HMV II; Shimadzu Corporation, Kyoto, Japan) was performed at a load of 50 g for a dwell time of 10 s [77]. Five indentations were made at each distance for each of six enamel samples for each composite. Healthy enamel hardness without biofilm attack was also measured as a control.

### 4.13. Statistical Analysis

All the data were checked for normal distribution with the Kolmogorov–Smirnov test. One-way and two-way analyses of variance with Tukey’s honestly significant difference (HSD) were performed for comparison. The statistical software SPSS 22.0 (SPSS Inc., Chicago, IL, USA) was used. *p* < 0.05 was considered to be significant.

## 5. Conclusions

This study showed that the DMAHDM + NACP composite inhibited enamel caries at the margins and protected enamel hardness in a saliva-derived biofilm-based secondary caries model for the first time. DMAHDM effectively disrupted the saliva-derived biofilm formation, viability and cariogenicity. Under biofilm acid attacks, the enamel hardness around the composite containing DMAHDM alone was 25% higher than that with control composite. With DMAHDM + NACP, the enamel hardness at the margins under biofilms was about 50% higher than the control. The NACP + DMAHDM composite exerted synergistic effects, with DMAHDM inhibiting biofilms and NACP neutralizing acids and remineralizing the enamel. These effects resulted in the greatest enamel hardness at the margins under biofilms that matched the hardness of healthy enamel.
